# Identifying “cities” of the world: a methodological approach, lexicon, and database for urban administrative units

**DOI:** 10.12688/openreseurope.21078.2

**Published:** 2026-04-13

**Authors:** Hiba Karam, Lalita Phatthanachaisuksiri, Vijay Palliyil, Meng Cai, Niklas Suhre, Eva Kassens-Noor

**Affiliations:** 1 Institute of Transport Planning and Traffic Engineering, Technische Universitat Darmstadt Fachbereich Bau- und Umweltingenieurwissenschaften, Darmstadt, Hesse, Germany

**Keywords:** Cities, Human Settlements, Database

## Abstract

Since 2015, there has not been a comprehensive database of cities across the world. While debates about what defines a “city” remain open in urban science, we center our efforts toward developing a replicable and transparent methodology to construct a globally valid dataset of cities, agglomerations, capitals, municipalities, and urban centers. Such an endeavor is both timely and necessary given that comparative research, governmental collaborations, and cross-national comparisons among urban areas yield valuable knowledge about human settlements to inform climate resilience and sustainability planning. Our approach is bottom-up and anchored in accessibility. We begin with Wikipedia, a dynamic, crowd-sourced platform, and cross-check its entries with an expert database based on census data. When discrepancies arise, we turn to official governmental websites to confirm information. We integrate these three sources into a harmonized database that includes every urban settlement worldwide with at least 50,000 residents (7,468 in total). For China, Brazil, France, Japan, India, Pakistan, Somalia and North Korea we apply a higher inclusion threshold of 100,000. Each entry lists country and city names and is complemented by a terminology lexicon explaining how different nations define and classify cities. This approach enables us to not only catalogue urban settlements but also to make visible how cities are constructed and recognized within diverse global national governance frameworks.

## Introduction

The question of “What is a city?” dates back to ancient philosophers grappling to define its form and function and the corresponding rights of its citizens ( Shahidipak, 2022). From a contemporary perspective, cities function more like dynamic ecosystems—interconnected spaces where residents, institutions, and economic actors interact and co-evolve ( Batty, 2013). Yet despite their centrality to everyday life and to research across disciplines, one fact remains constant: cities continually reshape themselves in response to changing agglomeration forces. Rapid urban population growth, compounded by nationally varying definitions ( Allen *et al.*, 2005; Gregory *et al.*, 2009; Parr, 2007) and divergent disciplinary classifications ( Caves, 2005; Chant & Goodman, 1999; Doxiadis, 1970; Paddison, 2001), has vastly outpaced the capacity of policymakers, industries, and researchers to maintain a timely, reliable, and verifiable global list of cities.

Even global entities, like the United Nations, have been struggling to answer that question since 1967 ( United Nations, 1969). Over the past five decades, the United Nations Population Division has been compiling a comprehensive demographic account of the global urbanization process, now recognized as the biennial “World Urbanization Prospects”. Yet despite these efforts, persistent inconsistencies in national definitions have prevented the creation of a unified global list. For example, the 2018 edition surveyed 233 countries and areas and found that 104 differed in both the number and criteria used to define their own cities ( United Nations, 2019).

Part of the challenge on defining what a city is lies in the conceptual instability of the term itself. The term city can be applied to “anything and everything” ( Caves, 2005) and is often used interchangeably with terms like urban area, metropolitan region, city proper, urban agglomeration, and capital ( UN-Habitat, 2020). This semantic fluidity complicates the task of producing a singular, globally valid definition ( UN-Habitat, 2020). A recent study brilliantly summarizes the various definitions of cities and their evolution ( Dong *et al.*, 2024).

Rather than defining what a city is, we focus instead on how to create a flexible methodology and verifiable list of cities. We will create a lexicon of settlements based on a new methodology, whereby users can flexibly identify cities as it suits their definition. It will fulfil two urgent needs: an up-to-date verifiable list of cities with their corresponding information (with populations of 50,000 and above ^1^), and an adaptable methodology that allows this lexicon to be expanded with lower population thresholds and continuously updated by anyone with access to the internet. This approach directly responds to UN-Habitat’s need for timely data and metrics ( UN-Habitat, 2020). to operationalize any local and/or global policy.

To the best of our knowledge (2024), five datasets ( Table 1) exist that list cities across the world. Two of the most comprehensive and academically grounded global datasets are the Atlas of Urban Expansion ( Angel *et al.*, 2016) and the latest public release of the GHSL Urban Centre Database ( Melchiorri *et al.*, 2024) New York University and UN-Habitat compiled the first dataset in 2010 as part of the *Atlas of Urban Expansion* project. This dataset identified 4,231 free-standing cities in 172 countries or territories with populations exceeding 100,000, using spatial criteria based on built-up areas and open spaces, rather than administrative boundaries ( Angel *et al.*, 2016). The second is the GHSL Urban Centre Database (GHS-UCDB R2024A/ “UCDB 2025”), a harmonised global inventory of urban centres derived from GHSL’s implementation of the Degree of Urbanisation (DoU) methodology ( Melchiorri *et al., *2024; Dijkstra *et al.*, 2021). Using consistent gridded population and built-up inputs (e.g., GHS-POP and GHS-SMOD), the latest release reports 11,422 quality-controlled urban centres worldwide ( Melchiorri *et al*., 2024).

**Table 1.** Existing city datasets and their limitations. Source: The authors.

Name of the dataset	Organisation	Date of the used data	Criteria	Number of cities	Shortcomings
1. Cities in the World	The OECD ^2^	2000–2015	Densely populated areas with at least 50,000 people and 1,500 inhabitants per square kilometre.	9,028	The dataset doesn’t include all countries in the world.
2. UN data Database	United Nations Statistics Division ^3^	1970–2023	The government decided on the list of cities since the data was collected based on questionnaires dispatched annually to national statistical offices.	4,968	The dataset is outdated because recent government lists have not been updated, e.g., Lebanon.
3. World Cities Database - simple maps	Free and paid versions ^4^	March 31, 2023	Based on authoritative sources from the USA.	44,691	It is non-governmental origin and solely determined by U.S. government agencies, potentially limiting its representativeness and inclusivity.
4. Geonames	Open source ^5^	November 10, 2023	All cities with a population > 1000 or seats of administrative divisions (ca 80.000).	141,184	It is a crowdsourcing scheme with an interface that allows everyone to sign in and edit the database, hence false information can be entered, and such information can remain undetected.
5. Urban Centre Database (GHS-UCDB R2024A/ “UCDB 2025” public release)	European Commission (JRC/GHSL) ^6^	Urban-centre boundaries reference year: 2025 (fixed boundary option); attributes reported 1975–2030	Derived from GHSL’s implementation of the Degree of Urbanisation (DoU) using gridded population and built-up layers. Urban centres are delineated on a 1 km grid based on population density and contiguity thresholds, independent of administrative boundaries.	11,422 (quality-controlled urban centres)	Because this method uses global grid density/contiguity thresholds, it delineates morphological/statistical urban centres that may differ from administrative city limits or functional urban areas.
6. The 2010 Universe of Cities	UN Habitat, New York University, Lincoln Institute ^7^	2010	Urban Extent, Built-Up, and Urbanized Open Space.	19,289	The dataset is outdated since it relies on data from 2010.

^2^ Source: Cities in the World | OECD

^3^ Source: UNdata | record view | City population by sex, city and city type

^4^ Source: World Cities Database | Simplemaps.com

^5^ Source: Geonames - All Cities with a population > 1000 — Opendatasoft

^6^ Source: GHS-UCDB R2024A - GHS Urban Centre Database 2025

^7^ Source: The 2010 Universe of Cities - Lincoln Institute of Land Policy

Both approaches produce harmonised urban footprints that may not fully coincide with administrative or statistical units. Satellite-derived Urban Expansion methods can capture urban form that extends across jurisdictional boundaries ( Angel *et al.*, 2016)., and grid-based delineations using population-density and contiguity thresholds may also differ from national administrative definitions ( Dijkstra *et al*., 2021). The first method identifies cities based solely on physical features, while the second relies on population data. Recognizing that cities also reflect other factors, several authors have proposed alternative ways to define their boundaries. For example, an exciting new approach to identifying cities has been developed in theory. Dong *et al.* (2024) proposed using cell-phone datasets, which capture the movement of people and serve as a proxy for the temporal and spatial connections that define urban life. The authors estimate that 7 billion cell-phone users create geolocated data across the world. This data can indicate people’s presence in specific areas and their movement between locations. They suggest that this method has the potential to become widely accepted for delineating cities. However, they do not address the challenge of individuals owning multiple phones or relying exclusively on wireless technology.

In summary, all three methods use an expert-driven, top-down methodological approach: the classification of cities from a top-down perspective, meaning they work with global governmental entities like the United Nations to decide what a city is. They require access to significant amounts of private and/or expensive data and need significant manpower, expertise, and resources to make a list of cities based on the three methodologies. In addition to these three, several other researchers have compiled similar lists (see Table 1), applying varying criteria for what constitutes a city and often capturing only a partial subset of cities worldwide ( Yap & Biljecki, 2023).

Despite numerous calls by geographers, demographers, and statisticians for internationally comparable definitions of urban areas and cities, the many different national definitions have not shown any tendency to converge to a common one. International comparability, especially in measuring urbanisation in a particular country, is likely not a major concern for national authorities ( Buettner, 2015).

In light of the persistent lack of consensus on what defines a city, we shift the focus from enforcing a universal definition to building a dataset grounded in how cities are defined by individual governments. We argue that the question is no longer *what* a city is, but *how* it evolves within its national context. Given the resource constraints and temporal limitations inherent in data collection, pursuing a single, fixed definition has become less relevant than developing a methodology that is adaptable over time. Our approach acknowledges that each government operates with its own criteria—criteria that inevitably shift in response to political, demographic, and spatial change. To accommodate this complexity, we offer a methodology, lexicon, and dataset that are responsive, transparent, and flexible tools designed to support researchers and policymakers alike.

## Method

To build our lexicon of cities, we draw on three foundational perspectives. Altshuler emphasizes the governance dimension of cities, highlighting the role of expert planners ( Warren, 1967); Jacobs portrays cities as dynamic, evolving ecosystems shaped by everyday human interaction ( Jacobs, 1961); and Corburn underscores the importance of local knowledge and participatory processes. Together, these views support a bottom-up understanding of urban classification ( Corburn, 2005).

In our lexicon, we identify each self-declared settlement alongside its official website, adopting the definitions and lists provided by local governments. This aligns with the principle of local governance ( Ndreu, 2016), where urban status is determined by municipal authorities rather than global institutions. This approach ensures contextual accuracy, official recognition, inclusivity, and currency. Our method triangulates three data sources: dynamic and crowd-sourced platform (i.e., Wikipedia ( Wikipedia, 2024), expert-curated databases City Population ( Brinkhoff, 2024a), and localized information (using websites of the human settlements) to create a final list.

Creating the lexicon and database contained six steps:

Step 1: To compile data on cities for the Lexicon, we simultaneously examined City Population ( Brinkhoff, 2024a) and Wikipedia ( Wikipedia, 2024). City Population, managed by Thomas Brinkhoff in Germany, is, to the best of our knowledge, the only website providing comprehensive global city information based on census results and official estimates ( Brinkhoff, 2024b). Wikipedia, being crowdsourced, allows for frequent updates by anyone. We compiled the data into an Excel file, grouping countries listed by the United Nation ( United Nations, 2025) into world regions as per Angel *et al.* (2016).

Step 2: We validated the list of cities in each country by initially referring to the region index pages in the City Population database and cross-referencing it with the country’s city list in Wikipedia. Subsequently, we conducted further validation by comparing the final list of country cities with the original data sources listed on both websites.

Step 3: If the lists from the two websites do not match, we check the government’s official documents for verification. Additional scrutiny involved checking the specific Wikipedia page of any extra city identified or other sources such as Google Maps. This process ensures the existence of the city. Moreover, if the source of information could not be verified on the City Population or Wikipedia websites, we only considered the list from the website with a verified reference source, and that source was further validated (e.g., Cambodia).

Step 4: If the years of the population numbers differ between the two websites, we select the latest available census or estimated population list. Moreover, during our data verification process, we may discover that the country has updated its city list on its official government website. However, this updated list may not have been reflected on the two websites we referenced. In such cases, we prioritized the list sourced directly from the government website, ensuring that we incorporated the most accurate and up-to-date information available (e.g., Thailand).

Step 5, for each city included in the final list, we documented its administrative level, identifying whether it was classified as a municipality, district capital, special-status city, or another type of administrative unit. This information was obtained either directly from the country’s official government list or from the city’s official website, depending on availability.

Finally, we used Google search to find the official website, alongside cross-referencing with the City Population and Wikipedia pages, ensuring that the website is indeed the city’s official site (See Workflow diagram 1). The websites of US cities were adopted from Cai *et al.* (2023).

**Workflow diagram 1.**
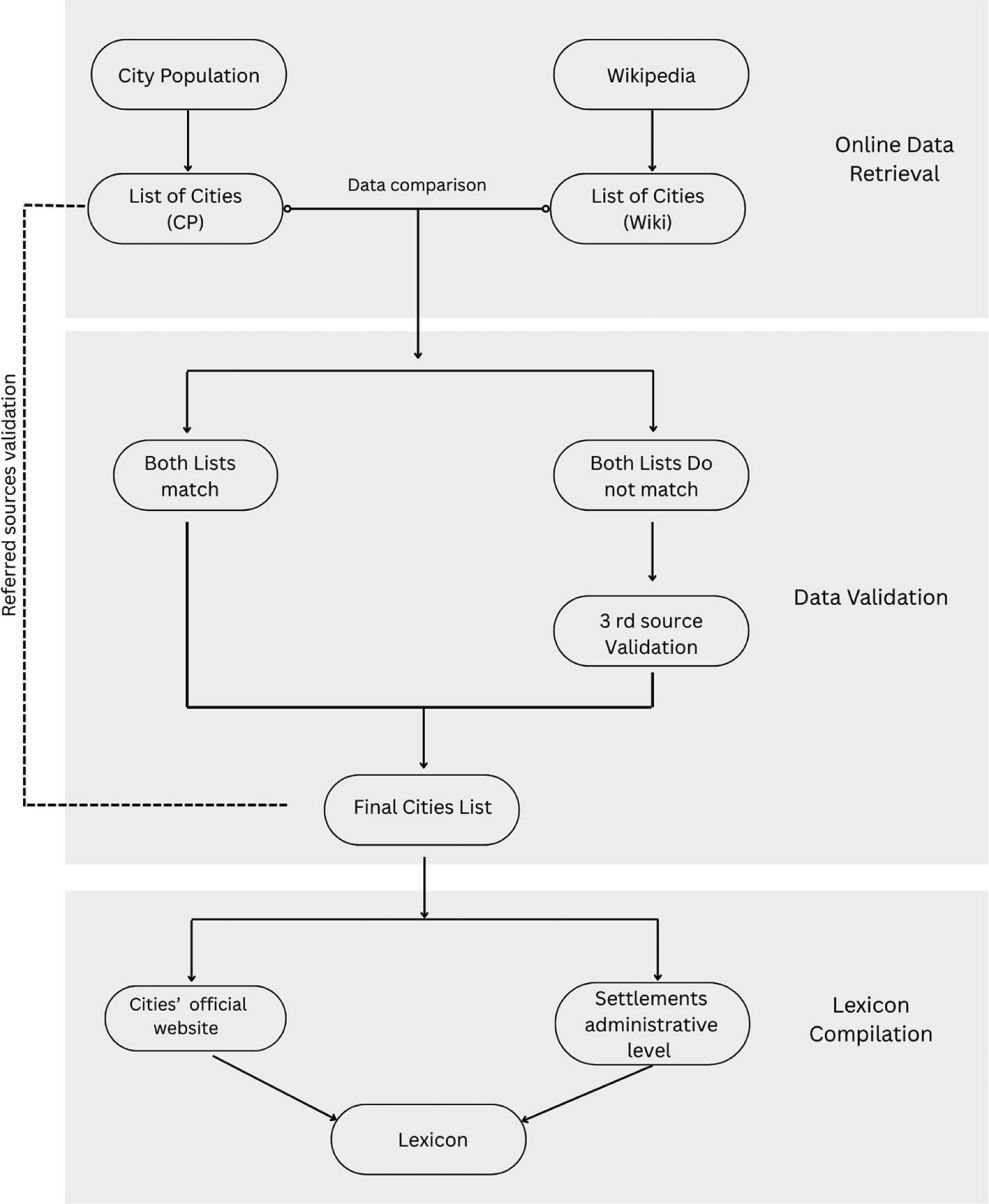
Illustrates the logic, operations, and decisions involved in the compilation and validation processes of compiling the lexicon. Source: The authors.

## Limitation

A core challenge in developing a global dataset through open-source platforms lies in their heavy reliance on predominantly Western-oriented sources, especially the English-language version of *Wikipedia.* This dependence raises valid concerns about representativeness and inclusivity—particularly in regions where *Wikipedia* content is censored or inconsistently developed across languages (see: Censorship of Wikipedia). To address this, we mitigated the bias by triangulating data from government websites and expert-curated databases.

Moreover, a further limitation is that, in countries where governments do not maintain up-to-date or accessible city lists, triangulation against official sources is constrained, and coverage may depend more on open platforms with uneven completeness. In such contexts, population-based settlement products could provide a complementary basis for future extensions.

### Future work

Integrating spatial data (e.g., from OpenStreetMap) is a promising avenue for future work to support mapping, geocoding, and spatial validation. In addition, the dataset is intended to enable iterative improvement by other researchers. We intentionally designed the workflow to be simple and transparent, so that anyone with internet access can reproduce the procedure and update the dataset.

## Data availability

Zenodo repository: “World Cities Lexicon – Urban Administrative Units and Population Data” https://zenodo.org/records/16419786 ( Karam *et al.*, 2025).

This project contains following underlying data:

• LexiconDataset_25072025.xlsx Contains major cities across world regions with their administrative classification, population data, official digital presence, and relevant geographic identifiers.

Data are available under the terms of the Creative Commons Attribution 4.0 International license (CC-BY 4.0).

### Contribution

We do not claim that our dataset is exhaustive, nor do we offer a silver bullet for the persistent challenge of defining what constitutes a city. Instead, we provide a replicable method to construct and validate a flexible, globally relevant dataset. We contribute to global comparability by clarifying how cities are defined and designated in different national contexts. Through the compilation of a lexicon that documents country-specific criteria, thresholds, and classification systems, the study equips users to better interpret the variation in how cities are recognized worldwide. This transparency enables more informed, purposeful sampling in cross-national research and policy development. In doing so, the lexicon promotes comparability not by enforcing sameness, but by rendering visible the diversity embedded in urban definitions making it accessible to researchers, practitioners, and policymakers alike.

A natural question arises: if an official list of cities, such as those from national census data, is available, why not use it directly as the foundation for the lexicon, given its presumed authority? However, locating and interpreting official government data is often challenging and time-consuming, especially when sources are available only in local languages unfamiliar to the researchers. In our dataset, when the lists from Wikipedia and City Population align, compiling the information becomes straightforward and efficient. But when discrepancies arise, cross-referencing the citations and references in Wikipedia or City Population can provide useful entry points, as either source may link to a more accurate or accessible government source.

## Footnotes

^1^
Except for China, Brazil, France, Japan, India, Pakistan, Somalia and North Korea we apply a higher inclusion threshold of 100,000 residents due to resource constraints.
