# Supplementary material for: Identifying “cities” of the world: a methodological approach, lexicon, and database for urban administrative units
Source: Open Res Eur. 2026 Apr 13;5:241. Originally published 2025 Aug 15. [Version 2] doi: 10.12688/openreseurope.21078.2 (PMC13103649; doi:10.12688/openreseurope.21078.2)
Supplement: Peer review history

# Reviewer report: Reviewer response for version 2

**DOI:** 10.21956/openreseurope.25458.r72204

**Published:** 2026-04-24

**Authors:** Johannes H. Uhl

**Affiliations:** 1 Joint Research Centre (JRC), Ispra, Italy

Dear authors, thank  you for addressing my previous suggestions. However, there are still some things that could be improved.

 1) The first sentence of the abstract states "Since 2015, there has not been a comprehensive database of cities across the world." - I am struggling with this statement. That implies there was such a dataset before 2015, but not after? Why 2015? This is very unclear. In the introduction, the authors mention the Atlas of Urban Expansion (Angel et al. 2016) and the Urban Centre Database (Melchiorri et al. 2024) - this contradicts that statement. I strongly suggest to make this statement clearer and less ambiguous, for example you could say "Creating a comprehensive database of cities across the world is a challenging task" or similar.

 2) In the intro, you mention the Atlas of Urban Expansion, but this dataset is then not included in Table 1. Is there a reason for that?

 3) There are a few further statements that seem a bit problematic to me:

 "Despite numerous calls by geographers, demographers, and statisticians for internationally comparable definitions of urban areas and cities, the many different national definitions have not shown any tendency to converge to a common one."

 --> The Degree of Urbanisation aims to tackle exactly this issue. There is no need that national definitions "converge to a common one". But there is a need that countries are able to disaggregate their statistical data into internationally comparable rural-urban classes. This process is ongoing and the number of countries that implement the Degree of Urbanisation as an additional classification (besides the national rural-urban definition) is steadily increasing (see https://doi.org/10.2785/3471430 for a recent summary)

 --> Moreover, there should be a reflection about national definitions of "cities" vs. "urban" / "rural" - many countries classify their territory into "urban" and "rural" (using statistical or non-statistical methods). The administrative delineation of a “city” may or may not coincide with “urban” definitions.

 --> I think it would be important to make clear that the administrative term "city" is often referring to an arbitrary geographic entity - in many cases, only representing the "core" of a city, that may or may not be the area where population concentrates. There are many examples where the "city" from an admin. point of view covers mainly the business district and surroundings, while the majority of the urban population resides in the outskirts where urban expansion occurred, and may not be included in the population counts used in this work. This is an important shortcoming of the method used herein.

 When discussing the approach of Dong et al. (2024), using cellphone data for urban delineations, the authors state “However, they do not address the challenge of individuals owning multiple phones or relying exclusively on wireless technology.”

- It is unclear what is meant by “relying exclusively on wireless technology”
- The problem of population overcounting by “individuals owning multiple phones” is likely a minor problem. The main issues with this approach are (a) that cellphone data are typically not open nor authoritative data, making the applicability of the approach dependent on data policies of individual countries or individual companies, and (b) data are not available for historical points in time. Measuring urban change in a consistent way is crucial to assess sustainable development and planning.

Lastly, I am still a bit concerned about the arbitrary change in population threshold for some countries. You state

 “Rather, due to resource constraints related to processing and validating very large numbers of settlements, we applied a higher inclusion threshold for China, Brazil, France, Japan, India, Pakistan, Somalia, and North Korea, limiting entries to settlements with at least 100,000 residents rather than the 50,000-threshold used elsewhere.”

- This arbitrary choice causes the settlement structure of these countries to be represented in a biased way and makes the dataset very difficult to use. I understand that part of this methodology consists of manual website checks, which may not be feasible in some cases – however, it would be a much better strategy to keep the threshold fixed for all countries, and omit the manual quality check in these countries, and flag these records as “not quality-checked”.

Are sufficient details of methods and materials provided to allow replication by others?

Partly

Is the rationale for creating the dataset(s) clearly described?

Partly

Are the datasets clearly presented in a useable and accessible format?

Yes

Are the protocols appropriate and is the work technically sound?

Partly

Reviewer Expertise:

Urbanization, geospatial analysis, human settlement modelling

I confirm that I have read this submission and believe that I have an appropriate level of expertise to confirm that it is of an acceptable scientific standard, however I have significant reservations, as outlined above.

# Reviewer report: Reviewer response for version 2

**DOI:** 10.21956/openreseurope.25458.r72203

**Published:** 2026-04-22

**Authors:** Milad Malekzadeh

**Affiliations:** 1 University of Helsinki, Helsinki, Finland

Thank you for responsing to my concerns. The authors have adequately addressed the issues I raised.

Are sufficient details of methods and materials provided to allow replication by others?

Yes

Is the rationale for creating the dataset(s) clearly described?

Partly

Are the datasets clearly presented in a useable and accessible format?

Yes

Are the protocols appropriate and is the work technically sound?

Partly

Reviewer Expertise:

Geography, GIS, Spatial Analysis

I confirm that I have read this submission and believe that I have an appropriate level of expertise to confirm that it is of an acceptable scientific standard.

# Reviewer report: Reviewer response for version 2

**DOI:** 10.21956/openreseurope.25458.r72391

**Published:** 2026-04-22

**Authors:** Yile Chen

**Affiliations:** 1 Department of Architecture, Faculty of Humanities and Arts, Macau University of Science and Technology, Taipa, Macau, China

Dear author, I can see that the article has been revised.

 Overall, I think it's very good, but there's one thing I don't quite understand: the article claims, "For China, Brazil, France, Japan, India, Pakistan, Somalia, and North Korea, we adopted a higher inclusion threshold, namely 100,000 cities," but the reasons for the different countries are not explained.

 Also, some hypothetical additions could be made to the applicable scenarios.

 In other words, besides supporting geographic mapping, are there any other application scenarios?

 China does not seem to include Hong Kong, Macau, and Taiwan. This is actually quite common, and I understand it. However, its limitations should be explained.

Are sufficient details of methods and materials provided to allow replication by others?

Partly

Is the rationale for creating the dataset(s) clearly described?

Yes

Are the datasets clearly presented in a useable and accessible format?

Yes

Are the protocols appropriate and is the work technically sound?

Yes

Reviewer Expertise:

urban planning

I confirm that I have read this submission and believe that I have an appropriate level of expertise to confirm that it is of an acceptable scientific standard, however I have significant reservations, as outlined above.

# Reviewer report: Reviewer response for version 1

**DOI:** 10.21956/openreseurope.22800.r59250

**Published:** 2025-11-05

**Authors:** Johannes H. Uhl

**Affiliations:** 1 Joint Research Centre (JRC), Ispra, Italy

Dear Authors, thank you for your hard work.

 Your effort is laudable, but this report needs further clarifications before it should be indexed

 1) I was unable to find a rationale for excluding China, Brazil, France, Japan, India, Pakistan, Somalia and North Korea from the lexicon. Could you clarify why you excluded these countries? It is particularly surprising e.g. for Brazil, which has high-quality geospatial data and classifications of their settlements.

 2) There are some misunderstandings regarding the Degree of Urbanisation, the Urban Centre Database, and the GHSL-SMOD datasets:

 - The Degree of Urbanisation (DoU) is a methodology, not a dataset. EC, UN-Habitat and others are working towards the implementation of this method in individual countries, which means that national statistical offices adopt the method to classify their territory acording to the Degree of Urbanisation method, enabling them to produce statistics for globally harmonised rural-urban classes using their own census data. The Degree of Urbanisation does not require that countries abandon their national definition of urban / rural, rather that they have an additional territorial classification allowing them to report internationally comparable urban/rural statistics. The "implementation" of the DoU means that individual countries adopt this method, and this implementation is an ongoing process, but this is not very relevant for your work, because of the following:

 - The European Commission Joint Research Centre (Global Human Settlement Layer project, GHSL) uses globally available census and population data to produce global gridded population data (GHS-POP R2023A). GHS-POP R2023A is available globally from 1975-2030. Based on GHS-POP, GHSL produced global gridded data of the Degree or Urbanisation (GHS-SMOD; "Settlement model") for the same spatio-temporal scope, also publicly available.

 Based on GHS-SMOD, GHSL extracted urban centres, which were enriched with a wide range of attributes, and are published as a global-scope dataset, called the Urban Centre Database (UCDB R2024A). In your report, you cite an old version of the UCDB. Please update this to the recent version of the UCDB.

 Moreover, GHSL classifies global administrative data according to the DoU method, published as the GHS-DUC (Degree of Urbanisation Classification).

 Hence, the fact that countries have not implemented the DoU method to their own census data does not hamper the applicability of the DoU method to the globe, since global gridded population data are available (GHS-POP) and the DoU method has been applied to that. The completeness / exhaustive property of the UCDB is not affected by the fact that the DoU implementation in countries is still ongoing.

 This needs to be clarified in the report.

 Moreover, the authors should clarify how exactly they searched for "cities" in Wikipedia. Often, the term "city" is used for an administrative unit (eg municipality), but in many cases, the "city" as defined by administrative institutions only comprises the center part of an urban settlement, while urban growth / sprawl / conurbation processes have caused the urban object (or: settlement) to extend far beyond the administrative borders of the "city". 

 I see the dataset is called "Urban Administrative Units" - This is actually a much more precise name. Your dataset is about urban administrative units, according to an arbitrary definition of "urban". It is not about "cities", and not about "settlements", it is about "Urban Administrative Units". I strongly suggest replacing "human settlements" by "Urban Administrative Units" in the title of this report.

 Another issue is the temporal heterogeneity of the population estimates reported in the proposed dataset, ranging from or beyond 2014 to 2023. Alongside with the lack of information for several important countries, I do not see how these population data could be useful for analytical purposes.

 I suggest to cross-compare the population numbers obtained in this database with the UCDB 2024A for the respective reference years.

Are sufficient details of methods and materials provided to allow replication by others?

Partly

Is the rationale for creating the dataset(s) clearly described?

Partly

Are the datasets clearly presented in a useable and accessible format?

Yes

Are the protocols appropriate and is the work technically sound?

Partly

Reviewer Expertise:

Urbanization, geospatial analysis, human settlement modelling

I confirm that I have read this submission and believe that I have an appropriate level of expertise to confirm that it is of an acceptable scientific standard, however I have significant reservations, as outlined above.

## References

[1] : Stats in the City – the GHSL Urban Centre Database 2025. *https://publications.jrc.ec.europa.eu/repository/handle/JRC139768* .2024;

[2] GHSL data package 2023. *https://op.europa.eu/en/publication-detail/-/publication/a1483a27-ed5d-11ed-a05c-01aa75ed71a1/language-en* .2023;

# Response

**Published:** 2026-03-25

**Authors:** Hiba Karam

**Affiliations:** Civil Engineering, Technische Universitat Darmstadt, Darmstadt, Hesse, Germany

Dear Johannes H. Uhl, Thank you very much for your careful reading of our manuscript and for your thoughtful and constructive comments. We greatly appreciate the time and effort you invested in reviewing our work. Your feedback has been very helpful in improving the clarity and quality of the paper. We have revised the manuscript accordingly, and below we respond to each comment in detail.

 1) I was unable to find a rationale for excluding China, Brazil, France, Japan, India, Pakistan, Somalia and North Korea from the lexicon. Could you clarify why you excluded these countries? It is particularly surprising e.g. for Brazil, which has high-quality geospatial data and classifications of their settlements.

 Thank you for raising this point, and we apologize for the confusion. We did not exclude these countries from the lexicon. Rather, due to resource constraints related to processing and validating very large numbers of settlements, we applied a higher inclusion threshold for China, Brazil, France, Japan, India, Pakistan, Somalia, and North Korea, limiting entries to settlements with at least 100,000 residents rather than the 50,000-threshold used elsewhere. We have revised the manuscript to explain this rationale more explicitly and to avoid any ambiguity that might suggest these countries were excluded.

 2) There are some misunderstandings regarding the Degree of Urbanisation, the Urban Centre Database, and the GHSL-SMOD datasets:

 - The Degree of Urbanisation (DoU) is a methodology, not a dataset. EC, UN-Habitat and others are working towards the implementation of this method in individual countries, which means that national statistical offices adopt the method to classify their territory acording to the Degree of Urbanisation method, enabling them to produce statistics for globally harmonised rural-urban classes using their own census data. The Degree of Urbanisation does not require that countries abandon their national definition of urban / rural, rather that they have an additional territorial classification allowing them to report internationally comparable urban/rural statistics. The "implementation" of the DoU means that individual countries adopt this method, and this implementation is an ongoing process, but this is not very relevant for your work, because of the following:

 - The European Commission Joint Research Centre (Global Human Settlement Layer project, GHSL) uses globally available census and population data to produce global gridded population data (GHS-POP R2023A). GHS-POP R2023A is available globally from 1975-2030. Based on GHS-POP, GHSL produced global gridded data of the Degree or Urbanisation (GHS-SMOD; "Settlement model") for the same spatio-temporal scope, also publicly available.

 Based on GHS-SMOD, GHSL extracted urban centres, which were enriched with a wide range of attributes, and are published as a global-scope dataset, called the Urban Centre Database (UCDB R2024A). In your report, you cite an old version of the UCDB. Please update this to the recent version of the UCDB.

 Moreover, GHSL classifies global administrative data according to the DoU method, published as the GHS-DUC (Degree of Urbanisation Classification). Hence, the fact that countries have not implemented the DoU method to their own census data does not hamper the applicability of the DoU method to the globe, since global gridded population data are available (GHS-POP) and the DoU method has been applied to that. The completeness / exhaustive property of the UCDB is not affected by the fact that the DoU implementation in countries is still ongoing.

 This needs to be clarified in the report.

 Thank you for this detailed information. We have revised our manuscript accordingly in the introduction section and updated Table 1 with the indicated new dataset. We understand that the Degree of Urbanisation is a method, and we rewrote our sentences to make that clearer.

 3) Moreover, the authors should clarify how exactly they searched for "cities" in Wikipedia. Often, the term "city" is used for an administrative unit (eg municipality), but in many cases, the "city" as defined by administrative institutions only comprises the center part of an urban settlement, while urban growth / sprawl / conurbation processes have caused the urban object (or: settlement) to extend far beyond the administrative borders of the "city". 

 Thank you for raising this important clarification. We used Wikipedia’s curated “Lists of cities https://en.wikipedia.org/wiki/Lists_of_cities” pages as a structured entry point to identify settlement across countries. These Wikipedia pages were used to retrieve and standardize city/settlement names and to provide reference links for user-led follow-up checks. We revised the wording in the methodology to make this clearer. Crucially, Wikipedia was not the primary determinant of inclusion in the final lexicon. The final list is derived from our core sources and harmonization procedure, with Wikipedia serving only a supplementary role (i.e., to support initial identification and facilitate verification).

   4) I see the dataset is called "Urban Administrative Units" - This is actually a much more precise name. Your dataset is about urban administrative units, according to an arbitrary definition of "urban". It is not about "cities", and not about "settlements", it is about "Urban Administrative Units". I strongly suggest replacing "human settlements" by "Urban Administrative Units" in the title of this report.

 Thank you for the comment. This is a great point. We agree and have changed the name accordingly.

 5) Another issue is the temporal heterogeneity of the population estimates reported in the proposed dataset, ranging from or beyond 2014 to 2023. Alongside with the lack of information for several important countries, I do not see how these population data could be useful for analytical purposes. I suggest to cross-compare the population numbers obtained in this database with the UCDB 2024A for the respective reference years.

 Thank you for this comment. We agree that the population figures are temporally heterogeneous and not suitable for direct analytical comparison without harmonization. We have clarified in the manuscript that they are provided mainly as contextual metadata rather than as a standardized analytical variable. We also added a limitation noting that, where official city lists are unavailable or outdated, coverage may depend more on open sources with uneven completeness, and that population-based settlement products could support future extensions.

# Reviewer report: Reviewer response for version 1

**DOI:** 10.21956/openreseurope.22800.r60429

**Published:** 2025-09-30

**Authors:** Milad Malekzadeh

**Affiliations:** 1 University of Helsinki, Helsinki, Finland

The rationale for creating this dataset is clearly articulated, and the need for such a resource is evident. However, while the motivation is well presented, there are existing datasets that cover similar ground, as listed by the authors in Table 1. Unfortunately, some of the information provided about these datasets is inaccurate. For example, I quickly reviewed the dataset from *simplemaps.com*. The basic version of their dataset is freely available, whereas the pro version contains substantially more information and is therefore not open access, though it is highly comprehensive. Additionally, the authors state that the city lists in previous datasets are outdated. However, according to the *simplemaps* website, their dataset was most recently updated in 2025. These discrepancies should be addressed and corrected in the revised manuscript.

 Regarding the contribution, this new dataset has clear value, and I commend the authors for their efforts in compiling and sharing it. That said, I have some concerns about the methodology. A widely used and authoritative source of spatial data for geographers, spatial analysts, and related fields is OpenStreetMap (OSM). Extracting a list of cities from OSM is straightforward by querying features labeled as “city.” While Wikipedia can certainly be a useful resource, it is a crowdsourced platform and, therefore, has limited reliability as a primary source for scientific data. Although the authors have attempted to ensure reliability by cross-checking information across multiple sources, it would strengthen the paper to include a comparison between the city list derived from OSM (while still crowdsourced, but the validity of its data has been studied over and over) and the dataset they have compiled. OSM has the additional advantage of providing spatial coordinates, which would make the dataset more readily applicable for quantitative spatial analyses.

 Furthermore, *simplemaps* states on their website that their dataset was “built from the ground up using authoritative sources such as the NGIA, US Geological Survey, US Census Bureau, and NASA.” It would be important to know whether the authors have investigated *simplemaps*’ methodology or contacted them directly to clarify their approach. If *simplemaps* also uses a bottom-up, authoritative method, then the novelty of the present dataset may be less clear. In such a case, the primary contribution of the authors’ work might shift toward providing an open and accessible version of similar data.

 Finally, for countries where governments do not maintain up-to-date city lists, or where Wikipedia lacks adequate information or cannot be fully trusted, approaches based on population data may offer a more comprehensive and reliable alternative. It would be helpful for the authors to discuss these issues explicitly and to justify their methodological choices in light of these considerations.

Are sufficient details of methods and materials provided to allow replication by others?

Yes

Is the rationale for creating the dataset(s) clearly described?

Partly

Are the datasets clearly presented in a useable and accessible format?

Yes

Are the protocols appropriate and is the work technically sound?

Partly

Reviewer Expertise:

Geography, GIS, Spatial Analysis

I confirm that I have read this submission and believe that I have an appropriate level of expertise to confirm that it is of an acceptable scientific standard, however I have significant reservations, as outlined above.

# Response

**Published:** 2026-03-25

**Authors:** Hiba Karam

**Affiliations:** Civil Engineering, Technische Universitat Darmstadt, Darmstadt, Hesse, Germany

Dear Milad Malekzadeh, We sincerely thank you for your thorough review of our manuscript and for your insightful comments and suggestions. We appreciate the opportunity to revise our work in response to your feedback.

  

1. The rationale for creating this dataset is clearly articulated, and the need for such a resource is evident. However, while the motivation is well presented, there are existing datasets that cover similar ground, as listed by the authors in Table 1. Unfortunately, some of the information provided about these datasets is inaccurate. For example, I quickly reviewed the dataset from *simplemaps.com*. The basic version of their dataset is freely available, whereas the pro version contains substantially more information and is therefore not open access, though it is highly comprehensive.

Thank you for highlighting this point. We agree that Simplemaps.com offers both a freely available basic version and a more comprehensive paid (“Pro”) version. We have revised Table 1 accordingly to reflect this distinction more accurately. Importantly, this clarification does not affect our broader argument regarding the dataset’s limitations for our purposes, as our concern relates primarily to the underlying data source and its characteristics (as discussed in point 6), rather than to the access model itself.

1. Additionally, the authors state that the city lists in previous datasets are outdated. However, according to the *simplemaps* website, their dataset was most recently updated in 2025. These discrepancies should be addressed and corrected in the revised manuscript.

Thank you for raising this point. We have changed the name from World Cities to World Cities Database – simplemaps to resolve any confusions as we did not characterize the simplemaps dataset as outdated (see Table 1 for our revised manuscript). Rather, our statement regarding outdated information refers specifically to (i) the UN Data Database (1970–2023) and (ii) the 2010 Universe of Cities, which is based on 2010 data, as reflected in Table 1.

1. Regarding the contribution, this new dataset has clear value, and I commend the authors for their efforts in compiling and sharing it. That said, I have some concerns about the methodology. A widely used and authoritative source of spatial data for geographers, spatial analysts, and related fields is OpenStreetMap (OSM). Extracting a list of cities from OSM is straightforward by querying features labeled as “city.”

Thank you for this helpful suggestion. We understand that OpenStreetMap (OSM) is a community-driven resource that draws on local knowledge, with contributors using aerial imagery, GPS devices, and field mapping to support accuracy and regular updates. Our dataset, however, is not intended to provide spatial geometries; rather, it focuses on compiling and harmonizing city lists and associated attributes. That said, we recognize the value of extending the dataset with spatial information, and we have incorporated this point into the manuscript’s “Future Work” section as a clear avenue for further development and the potential integration of spatial data.

1. While Wikipedia can certainly be a useful resource, it is a crowdsourced platform and, therefore, has limited reliability as a primary source for scientific data.

Thank you for this important point. We acknowledge that Wikipedia is a crowdsourced platform and therefore has limitations as a primary scientific source. In our dataset, however, it is not used as a principal data source, but rather as a practical starting point for users seeking to further update the city list. We have explicitly addressed this concern in the manuscript’s Limitations section, where we discuss the uneven geographic and linguistic coverage of open-source platforms, including the disproportionate reliance on English-language content, and the resulting implications for representativeness, particularly in contexts of censorship or uneven coverage across language editions. As noted there, we mitigate this limitation by triangulating entries against government websites and expert-curated databases. In addition, we clarify that when discrepancies arise between lists, cross-referencing the citations and references provided in Wikipedia or City Population may offer useful entry points, as either source can lead users to a more accurate or accessible government source.

1. Although the authors have attempted to ensure reliability by cross-checking information across multiple sources, it would strengthen the paper to include a comparison between the city list derived from OSM (while still crowdsourced, but the validity of its data has been studied over and over) and the dataset they have compiled. OSM has the additional advantage of providing spatial coordinates, which would make the dataset more readily applicable for quantitative spatial analyses.

Thank you for your suggestion. We aim to make differences across city lists transparent in order to support more informed comparative case selection, rather than to provide a ready-to-use spatial dataset. However, we have added the integration of spatial data as a clear avenue for future work.

1. Furthermore, *simplemaps* states on their website that their dataset was “built from the ground up using authoritative sources such as the NGIA, US Geological Survey, US Census Bureau, and NASA.” It would be important to know whether the authors have investigated *simplemaps*’ methodology or contacted them directly to clarify their approach. If *simplemaps* also uses a bottom-up, authoritative method, then the novelty of the present dataset may be less clear. In such a case, the primary contribution of the authors’ work might shift toward providing an open and accessible version of similar data.

Thank you for pointing this out. Yes, we reviewed Simplemaps’ methodology. Simplemaps describes its World Cities Database as being compiled from “authoritative sources” such as the National Geospatial-Intelligence Agency, the U.S. Geological Survey, and the U.S. Census Bureau. Its FAQ further states that, in response to the question “What counts as a city/town?”, the database includes “any populated place in the world as determined by U.S. government agencies.” This suggests a top-down inclusion criterion rooted in institutional classification, which is conceptually different from our bottom-up procedure. Accordingly, we do not position our contribution as providing “better” data. Rather, we present a dataset designed for a different research purpose: to make differences across city lists transparent and thereby support more representative and reproducible comparative case selection, based on explicit inclusion criteria and traceable, ground-level evidence rather than a single authoritative determination of what qualifies as a “populated place.”

1. Finally, for countries where governments do not maintain up-to-date city lists, or where Wikipedia lacks adequate information or cannot be fully trusted, approaches based on population data may offer a more comprehensive and reliable alternative. It would be helpful for the authors to discuss these issues explicitly and to justify their methodological choices in light of these considerations.

Thank you for your comment. This is clearly one of our work’s limitations. We have included it in the limitation section in our revised manuscript.
